# Pneumonia

**Published:** 1855-07

**Authors:** 


### EDITORIAL DEPARTMENT.

*Pneumonia.*— Our readers will notice, in our report of the proceedings of the Buffalo Medical Association, the histories of several cases of pneumonia. We are informed that this disease has been more than usually prevalent in Western New York this spring. This fact, together with some comments excited by the republication of a clinical lecture by Prof. Metcalfe in our last number, have induced us to say a word or two on the treatment of pneumonia, editorially. We do so with the more freedom, as we are confident that our friends in the association will not attribute to us any desire to be hypercritical, or to attack either them or their treatment.

Indeed, were this a question to be referred to authorities, we should find the practice of free and repeated bloodletting in pneumonia, sanctioned by the large majority of standard writers, and, to all appearance, by the results of successful treatment. Here, for instance, are a number of cases reported, in which bloodletting was practiced. In all of them the result was favorable, and it is altogether probable that the statistics of venesection in this disease, were they collected, would show a very fair average of success.

Admitting this to be a fact, it by no means follows that the whole question is settled. Other sets of facts, equally well authenticated, are in existence, going to prove that other forms of treatment are equally successful. In our own practice — and we have treated not a little pneumonia — we have never bled a case, and looking back now over a period of ten years, we cannot recall a case where we regret the omission of this remedy. During the period alluded to, we have had unfavorable results in three cases. One of these was a gentleman aged 73 years, with typhoid tendencies from the outset. Another was a lady of about the same age, with double pneumonia, and, as we thought at the time, not a proper subject for depletion under any circumstances. Both of these cases proved fatal. In the third case our patient was a boy of sixteen, and we had pleuritic abscess following; the result of the case, however, being a cure. In this latter case, if in any, we should have bled, as the pleuritic complication was very serious, though not prominent in the form of pain.

Now in all our other cases we have been fortunate enough to see our patients recover speedily, and without unpleasant consecutive results in the way of impaired constitution, abscess, or cough.

The practice of venesection in pneumonia rests on a supposed necessity for its use, originating in the idea that pneumonia, unless speedily controlled, is uniformly a dangerous disease, implicating the life of the patient. Dr. Metcalfe—and he is a very careful and judicious observer—insists strongly that this idea is erroneous, that the tendency is not toward death, but rather toward recovery, and that a mild expectant treatment is all that is necessary in the usual form. At the same time we do not understand him to reject the lancet in those severer cases implicating the greater extent of the organ.

It is only necessary to refer to the evidence adduced by Dr. Metcalfe, as its very recent publication in our pages must render it familiar to our readers. But if we look carefully at it, and at the results of all other forms of treatment, we shall find that for all of them about an equal success is claimed. The English treatment by calomel, antimony, and the lancet; the *extractum graminis* of Skoda; the chloroform inhalations of Varrentrapp; the rest and diet of Dietl; the antimonial treatment so common in this country, the acetate of ammonia of Dr. R. B. Todd, all have their friends and advocates, each satisfied with his own routine, and indisposed to change.

There is but one rational explanation for this. A man does not wed himself to an unsuccessful treatment and remain satisfied. We thoroughly believe that no accusation of grave fatality can be brought against any one of these various methods, for the simple reason that the disease is not a dangerous one, and that the patients are usually young and vigorous enough to bear a little, or a good deal, of depletion without permanent or obvious injury.

But if we adopt these conclusions we must also adopt the milder forms of treatment. A bloodletting, a course of antimony or calomel, if unnecessary, are wrong. They must more or less implicate the convalescence and subsequent condition of the patient.

We have confined these remarks to the relative success of different forms of management, without reference to diversities in the method of bloodletting, because it seems to us that before we consider whether to bleed *pleno rivo*, repeatedly, early or late, before or after hepatization, we should first decide whether or not to bleed at all. If the disease is self-limited, not dangerous, having a tendency toward recovery, it is certainly better to trust nature than the lancet.

